# Phosphorus-Loaded Biochar-Assisted Phytoremediation to Immobilize Cadmium, Chromium, and Lead in Soils

**DOI:** 10.1021/acsomega.3c07433

**Published:** 2024-01-09

**Authors:** María F. Serrano, Julián E. López, Nancy Henao, Juan F. Saldarriaga

**Affiliations:** † Department of Civil and Environmental Engineering, Universidad de los Andes, Carrera 1Este #19A-40, 111711 Bogotá, Colombia; ‡ Facultad de Arquitectura e Ingeniería, Institución Universitaria Colegio Mayor de Antioquia, Carrera 78 #65-46, 050034 Medellín, Colombia

## Abstract

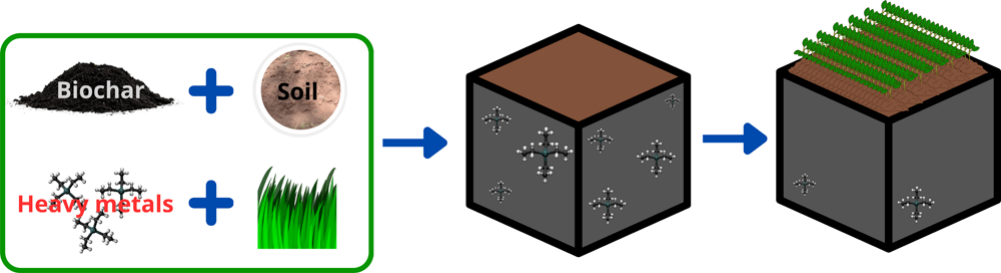

Soil contamination with heavy metals (HM) poses significant challenges to food security and public health, requiring the exploration of effective remediation strategies. This study aims to evaluate the remediation process of soils contaminated with Cd, Cr, and Pb using *Lolium perenne* assisted by four types of biochar: (i) activated coffee husk biochar (BAC), (ii) nonactivated biochar coffee husk (BSAC), (iii) activated sugar cane leaf biochar (BAA), and (iv) nonactivated biochar sugar cane leaf (BSAA). Biochar, loaded with phosphorus (P), was applied to soils contaminated with Cd, Cr, and Pb. *L. perenne* seedlings, averaging 2 cm in height, were planted. The bioavailability of P and heavy metals (HM) was monitored every 15 days until day 45, when the seedlings reached an average height of 25 cm. At day 45, plant harvesting was conducted and stems and roots were separated to determine metal concentrations in both plant parts and the soil. The study shows that the combined application of biochar and *L. perenne* positively influences the physicochemical properties of the soil, resulting in an elevation of pH and electrical conductivity (EC). The utilization of biochar contributes to an 11.6% enhancement in the retention of HM in plant organs. The achieved bioavailability of heavy metals in the soil was maintained at levels of less than 1 mg/kg. Notably, Pb exhibited a higher metal retention in plants, whereas Cd concentrations were comparatively lower. These findings indicate an increase in metal immobilization efficiencies when phytoremediation is assisted with P-loaded biochar. This comprehensive assessment highlights the potential of biochar-assisted phytoremediation as a promising approach for mitigating heavy metal contamination in soils.

## 1 Introduction

Environmental deterioration is one of the major issues facing society today. Some reports reveal a sharp increase in the loss of agriculturally valuable soils and water resources.^1,2^ Around the world, soil pollution with heavy metals (HMs) is causing significant concern and is one of the major threats to public health and food safety.^3^ Although it has been noted in the literature that anthropogenic activities have contributed to increasing the level of contamination of the environment with heavy metals (HMs), their presence in the environment may be attributable to natural causes. There is a large concentration of these in the soil and water because of industrialization, mining, population growth around the world, and massive waste generation.^4^

The presence of HMs in the environment is an important issue that must be addressed since they cannot be degraded and, therefore, are considered stable and persistent pollutants when deposited into the environment.^5^ Furthermore, they can be accumulated in ionic form and remain in organisms for long periods of time.^6^ The HMs’ toxicity depends on speciation, which is influenced by the physicochemical properties of the soil and the organic and inorganic ligands present.^7^ In North America, it is estimated that approximately 600 000 ha of vacant land have been contaminated with HMs.^8−10^

The presence of metals in the soil such as cadmium (Cd), chromium (Cr), lead (Pb), mercury (Hg), and nickel (Ni) have increased in the past decade, posing serious threats to humans, animals, and plants.^4^ Among the most toxic metals, Cd stands out due to its high mobility and persistent nature.^4^ Cd can accumulate in the kidneys, affecting the filtration mechanism and causing damage to them. It also causes problems in the immune central nervous system and possible DNA damage or the development of cancer.^11^ Pb is another heavy metal that is highly toxic to humans, due to its strong affinity for the SH-sulfide groups present in metabolic enzymes.^12^ In the human body, Pb can cause damage to the urinary, nervous, reproductive, and immune systems (EPA, 2022). Cr has been identified as an inhalation carcinogen. Some studies suggest that ingestion of Cr(VI) can lead to various health implications, such as cancer in the gastrointestinal tract.^13^ Concentrations of metal elements, namely, Cd, Cr, and Pb, have been documented in agricultural and industrial soils across various countries, including Holland, Brazil, Spain, and China. The reported concentrations for Cd range from 0.43 to 0.8 mg/kg, for Cr vary between 16 and 100 mg/kg, and for Pb, concentrations fall within the range of 13 to 85 mg/kg in these specified regions.^14−18^ Furthermore, on a global scale, the reported ranges for metal concentrations exhibit broader variability, covering from 0.06 to 11 mg/kg for Cd, 7 to 221 mg/kg for Cr, and 10 to 85 mg/kg for Pb. These comprehensive findings shed light on the diverse levels of metal contaminants in agricultural and industrial soils across different geographic locations, providing valuable information for environmental assessments and management strategies.^14−18^

The elimination of HM contamination in soil can present difficulties due to persistence, harm, and resistance to biodegradation. Likewise, HMs can accumulate and increase concentration throughout the food chain, causing toxic effects on human health.^19^ Consequently, it is essential to reduce or eliminate these risks to preserve both the environmental health of humans and that of living beings. Different technologies have emerged to remediate soils, among which are physicochemical strategies such as soil washing, electrokinetic remediation, chemical oxidation, remediation with nanomaterials, electrodialysis, leaching, stabilization, and landfilling.^20−22^ However, these methods are often expensive due to the high price of off-site waste disposal, not to mention the secondary environmental problems they can cause.^23^ An alternative is phytoremediation, a highly effective and promising technology to decontaminate, in this case, HM-contaminated matrices such as soil by natural means.^24−26^ This biological technology has established itself as an attractive alternative for the treatment of environmental pollution.^27,28^ Although there are several specific definitions, the basic definition involves the cultivation of plants in a contaminated substrate in order to eliminate, transform, or stabilize the environmental pollutants present.^29,30^ This method has been successfully used for the remediation of HMs in contaminated soils because it is a more effective and economical technique than other engineering techniques such as excavation, soil washing, incineration, solidification, and others.^31^ However, poor soil physicochemical properties, such as low nutrient content and extreme pH, combined with high concentrations of metals and metalloids, can prevent soil vegetation from growing. Therefore, assisted phytoremediation, which consists of adding amendments to the soil to improve conditions for plant growth, is often required. One of the organic amendments that has received the most attention in recent years is biochar.^23^

Biochar is a carbonaceous and porous material produced by the pyrolysis of organic compounds. Biochar has unique chemical and physical properties such as being pH neutral or alkaline, great surface area for the sorption of most metals, the presence of ash, a higher carbon content, and the ability to immobilize toxic heavy metals. Therefore, it can be successfully used as an immobilizer for various HMs.^31−33^ The high alkalinity and pH of biochar could be the cause of the reduced bioavailability of metals and is associated with the increased rate of precipitation in the soil when modified by the biochar process.^34,35^ Previously, it was used to improve the physical, chemical, and biological properties of soil to enhance crop production.^36^ Combining biochar with a phytoremediation method can significantly increase the effectiveness of HM remediation by improving biomass production and plant growth by 10%. This is due to biochar properties such as water holding capacity (WHC), cation exchange capacity (CEC), and high pH, which translates into greater nutrient availability and therefore higher biomass production.^31^ The sorption process of biochar primarily relies on the existence of cations that engage with the soil’s elements, effectively immobilizing them. Elements like CO_3_^2–^, SO_4_^2–^, and PO_4_^3–^ can interact with HMs inside the soil, creating intricate compounds that subsequently lead to precipitation.^37,38^ Phosphorus (P) is one of the most limiting nutrients in soils due to its fixation in the soil. It has been shown that P-loaded biochar is a good alternative because of its adsorption capacity, which makes it a good slow-release P amendment.^32,39−41^

Biochar also helps to reduce greenhouse gas emissions from agricultural residues. This is because it has a high recalcitrant carbon content, which contributes to increased soil carbon sequestration.^31,42,43^ In Colombia, more than 71 million tons/year of crop residues are produced, among which sugar cane and coffee stand out, of which only 17% is used.^44^ The waste produced in the coffee industry is 5 million tons/year between pulp, husks, and stalks,^45,46^ while the waste generated in the sugar cane industry in the case of leaves is estimated at 9 million tons/year.^44^ Utilizing this waste via pyrolysis to produce biochar with suitable physical and chemical attributes for enhancing and rehabilitating soils, including the HM containment, significantly bolsters the circular economy and environmental sustainability.^25,32,33^

In order to better understand the behavior of the combination of amendments in phytoremediation processes involving *L. perenne* for metals, such as Cr, Cd, and Pb, the removal efficiencies of two types of biochar from two different biomasses (coffee and sugar cane leaves) have been assessed. The aim was to evaluate, on a laboratory scale, the phytoremediation of different soils contaminated with Cd, Cr, and Pb using *L. perenne* assisted with a slow-release amendment of P-loaded biochar. Four types of amendments were evaluated: (i) activated (BAC) and (ii) nonactivated (BSAC) coffee husk biochar and (iii) activated (BAA) and (iv) nonactivated (BSAA) sugar cane leaf biochar. The novelty of this study focuses on understand how an amendment like P-loaded biochar can enhance the phytoremediation process involving a species such as *L. perenne*, renowned for its hyperaccumulation of certain metals and its susceptibility to this amendment. Additionally, it demonstrates the efficacy of biochar in immobilizing these metals, showing noticeable alterations in essential physicochemical properties across all of the evaluated treatments over a span of 45 days.

## 2 Results and Discussion

### 2.1 Tracking of pH, EC, P Bioavailability, and HMs

#### 2.1.1 pH

Soil samples to which 1% biochar (BAC, BSAC, BAA, or BSAA) was incorporated had a significant effect on pH compared to samples without biochar (blanks), increasing pH by 13.9% (*p* < 0.05). This increase corroborates that the addition of biochar to the soil raises the pH of the soil, which helps to immobilize metals.^47^ This may be due to reasons such as the alkaline pH of biochar, which causes a liming effect that raises the pH of the soil.^48^ Also, it may be associated with biochar causing ash accumulation and releasing cations and, therefore, proton-consuming reactions in the soil that decrease soil acidity.^49^ Soil pH is known to be an important parameter that influences many soil properties and affects the behavior of nutrients and HMs. For example, cations are more available at acidic pH, while the mobility of anions is higher at alkaline pH.^50^

**Figure 1:**
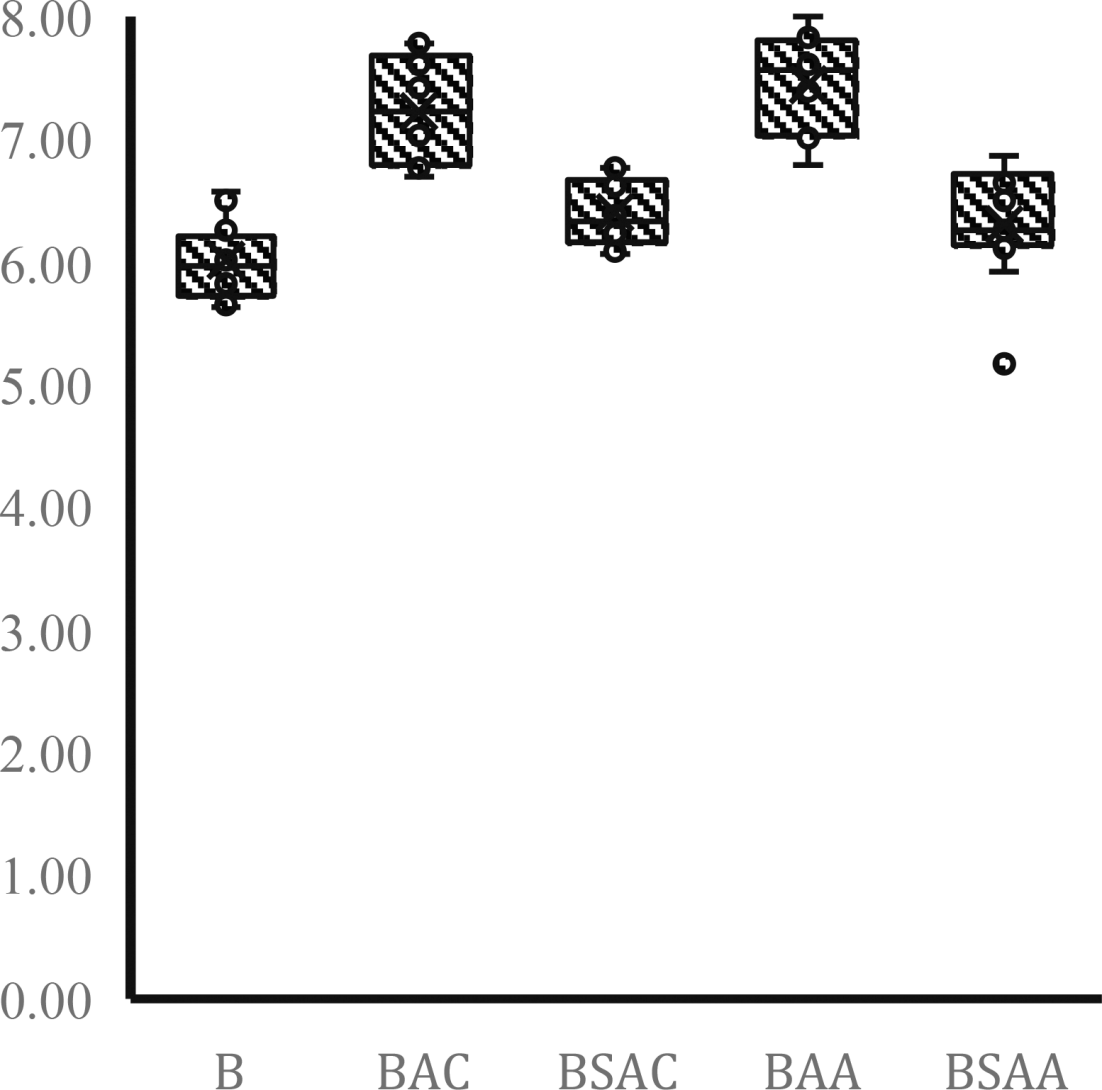
Behavior of pH over time grouped by type of treatment (*p* < 0.005).

It was not found that Cd, Pb, and Cr had significantly different effects with respect to the pH in the samples. Therefore, it was found that at least the biochars BAC and BAA contributed to the increase in soil pH in ratios of 19.29% and 15.77%, respectively, compared to the other samples. Activated biochar has a significant effect on the pH of the samples as shown in Figure 1. This could be since the activation was carried out with KOH (strong base). Various authors have reported that the increase in pH is due to the increase in K^+^ concentrations present in biochar,^51^ which is consistent with the results of this study.

In order to specifically determine in which cases the pH was increased, the averages over time were taken for each of the biochar types according to the type of soil contaminant. For soil contaminated with Cr, Cd, and Pb, it was found that at least one type of biochar had a highly significant effect on pH that was different from the rest.

Based on Figure 1 and the statistical analysis, significant differences can be observed between the different types of biochar, from which it can be highlighted that (i) the pH of samples 1.2 and 1.4 were on average 17.65% higher than the pH of the blank and samples 1.3 and 1.5; (ii) the pH of samples 2.2 and 2.4 was on average 17.86% higher than the pH of the blank; and (iii) the pH of samples 3.2 and 3.4 was 24.93% higher than the pH of the blank and samples 3.3 and 3.5. In summary, the activation of both coffee husk and sugar cane leaf biochar had a significantly greater effect on pH in soils contaminated with the three types of HM.

#### 2.1.2 Electrical Conductivity (EC)

Soil samples incorporated with 1% biochar (BAC, BSAC, BAA, or BSAA) had an impact on EC compared to samples without biochar (blanks), resulting in a 40.57% increase in EC. The influence of biochar on EC might be linked to the production processes, as higher pyrolysis temperatures have been demonstrated to yield biochar with enhanced properties, including EC, potentially enhancing soil quality, as observed in this study.^40,52−54^ Additionally, various studies in the literature have noted that the increase in pH and EC in biochar-modified soils correlates with their ash content.^55,56^ Some authors found a positive correlation (*r* = 0.75) between EC, biochar ash content, and concentrations of Mg, Ca, K, and P (*r* ≥ 0.4).^51^ It could be suggested that the rise in EC between control samples and treatments might be associated with the pyrolysis temperature used for biochar production (500 °C), coupled with the enrichment of biochar with P, potentially elevating the salt content. The EC values fell within the nonsaline range of 0 to 2000 μS_cm_^–1^, demonstrating that P-enriched biochar could not only enhance soil properties but also promote phytoremediation processes in plants such as *L. perenne*.

**Figure 2:**
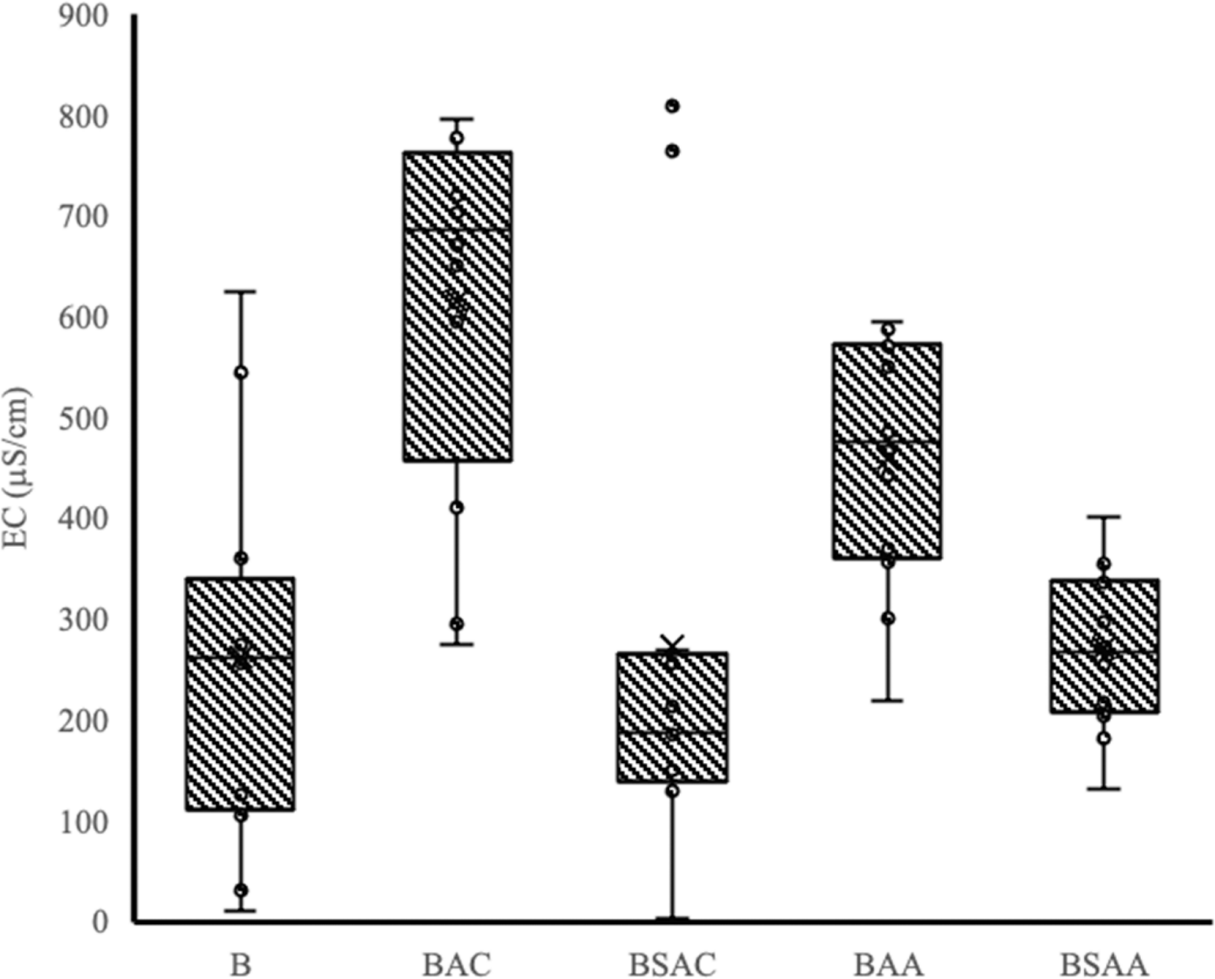
EC values grouped by type of treatment.

At least one HM was not found to have a significantly different effect on the EC of the samples; therefore, it is concluded that the HM does not affect the EC of the samples. Consequently, it was grouped by biochar type, including the blank, and it was found that at least one type of biochar had a significantly different effect on EC. BAC contributed to a 2.31-fold increase in soil EC compared to the other biochar tests (Figure 2). In this case, activation and biomass type contributed to the significant increase in soil EC. This could be because activation has been done with KOH (strong base). Various authors have reported that the increase in EC is due to the increase in K^+^ concentrations present in biochar,^51^ which is consistent with the results of this study.

In order to specifically determine in which cases the EC was increased, averages over time were taken for each of the biochar types tested according to the type of soil contaminant. For Cr and Pb contaminated soils, at least one type of biochar was found to have a significant effect on EC different from the rest. For Cd-contaminated soil, at least one type of biochar was not found to have a significantly different effect on EC compared with the rest. Therefore, it can be assumed that there is no difference in the EC in Cd-contaminated soil.

Based on Figure 2 and the statistical analysis, significant differences can be observed between the different types of biochar, highlighting that (i) the EC of sample 1.2 was on average 2.41 times higher than the EC of the blank, sample 1.3, and sample 1.5; (ii) the EC of sample 1.4 was on average 1.07 times higher than that of the blank and sample 1.3; and (iii) the EC of sample 3.2 was on average 2.64 higher than the EC of the blank, sample 3.3, and sample 3.5. Therefore, it could be demonstrated that for Cr-contaminated soil, biochar activation contributed to the increase in EC compared to the control sample and nonactivated biochar soils, and for Pb-contaminated soil, coffee husk biochar activation was associated with the increase in EC.

#### 2.1.3 Evaluation of Heavy Metal (HM) Bioavailability over Time

**Figure 3:**
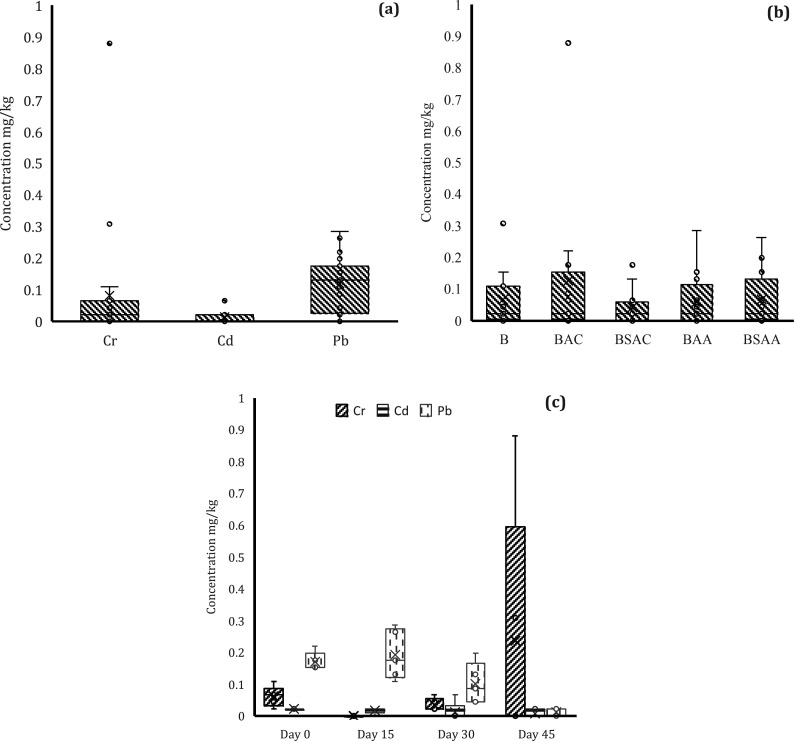
Bioavailability of MHs grouped by (a) type of MH, (b) type of treatment, and (c) day of follow-up (*p* < 0.05).

For the metal availability analysis, averages over time were taken for each of the biochar types according to the type of soil contaminant. For the three soil types (Cr-, Cd-, and Pb-contaminated), at least one type of biochar was found to have no significantly different effect on metal availability. Based on the above, it was grouped by HM (Figure 3a), and the bioavailability of HMs in soil was not found to be different among the three types of contaminated soils. Therefore, it was grouped by biochar type, and it was not found that at least one type of biochar had a significantly different effect on the bioavailability of HMs (Figure 3b).

The bioavailability of the three HMs was analyzed over time independently of the type of treatment used (Figure 3c). For Cr-contaminated soils, no significant differences were found between the Cr concentrations. Nevertheless, it can be noted that for the Cr-contaminated soil, the concentration increased on day 45. This increase is related to samples 1.1 and 1.2. In sample 1.1, this could be due to the absence of biochar, and in sample 1.2 it could be due to the use of P-loaded biochar. For although they are a promising tool for immobilization of HMs (e.g., Pb, Zn, Cu and Cd), as they are a phosphorus nutrient supplement to improve soil properties, thanks to the processes of precipitation and complexation of phosphate/^–^OH/^–^COOH with HMs.^57^ According to Xu et al., special care should be taken when applying P-loaded biochar to treat Cr-contaminated soils because of the potential for negative feedback. This may be due to the fact that P is an As/Cr analog and can compete for binding sites when added to soil.^59^ It is important to consider whether the modified components present in biochar-based materials conflict with the intrinsic properties of HMs. For Cr-contaminated soils, no significant differences in Cr concentrations were found, and it can be observed that the bioavailability of Cr in soils was less than 0.1 mg/kg on all monitoring days. In the case of Pb-contaminated soil, a significant effect of time on the bioavailability of Pb in soil was found, with Pb concentrations at day 15 > day 30 > day 45. In this case, the expected trend of decreasing bioavailability of MH over time can be observed. This could be due to the increase in pH or the incorporation of P-loaded biochar, which according to Gao et al.^57^ favors Pb precipitation by directly and indirectly increasing soil pH and the amount of available P. Consistent with the above, it is known that under alkaline pH conditions, for example, Pb and Cd tend to form hydroxide or carbonate precipitates. Consequently, the increase in pH induced by biochar may contribute to the immobilization of Pb and Cd in the form of these precipitates.^50^ Therefore, this may be one of the reasons for the absence of Cd and Pb bioavailability at the end of follow-up in the present study.

Low levels of HMs are generally observed over time. It can be concluded that soil remediation can be achieved with either type of raw material used to produce biochar and that there are no significant differences between activated and nonactivated biochar. However, special attention should be paid to the addition of P-loaded biochar to Cr-contaminated soils as the bioavailability of P increased at the end of the monitoring period, although the concentration remained at a low level. The low bioavailability of HMs in the soil seems to indicate that the mechanism of precipitation or phytoremediation (the content of HMs could be in the plant organs of *L. perenne*) would be favored.^60−62^

In the results of this study, a soil stabilization process was achieved, as evidenced in Figure 3, thanks to the addition of biochar loaded with P. An immobilization of metals is shown at day 45 (Figure 3c). Due to the low bioavailability of metals such as Cd and Pb, this may be due to, as has been documented by other authors, processes such as complexation, precipitation, and redox reactions that could have occurred during the 45 days of treatment.^63,64^ However, it is observed that unlike Cd and Pb, in the case of Cr, an increase in its bioavailability is observed at day 45. Due to the alterations that the biochar could have produced in the physicochemical properties of the soil, increasing the availability of metals (loids) or polycyclic aromatic hydrocarbons (PAHs) is typical of pyrolytic degradation.^64−67^ It is necessary to continue investigating the processes associated with the remediation of Cr and the application of biochar loaded with P, because it is evident in this study that in the first days there is no bioavailability of this metal, but after some time, it is clear if there is a high bioavailability. Alternatively, if it is required, this biochar is combined with organic amendments that have been extensively shown to be effective in lowering the bioavailability of metals like Cr over an extended period of time.^53,54,68^

#### 2.1.4 Evaluation of Phosphorus (P) Bioavailability over Time

**Figure 4:**
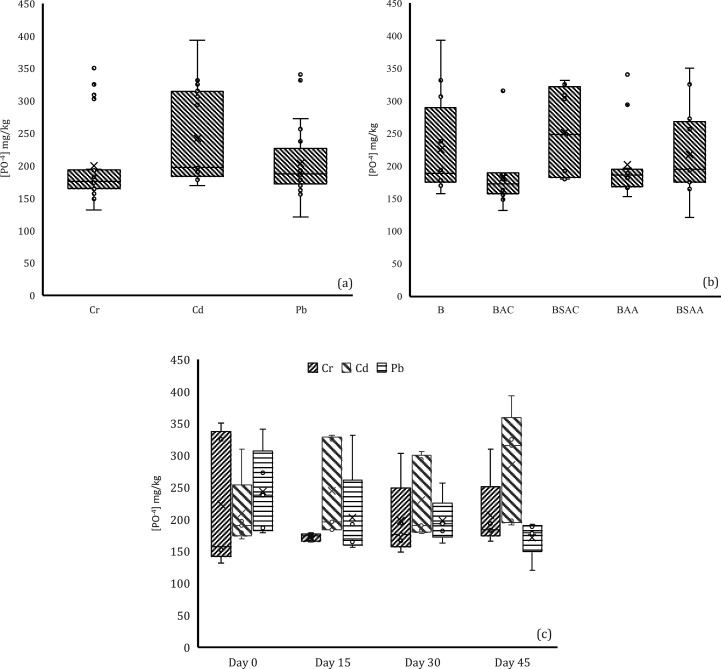
P bioavailability grouped by (a) HM, (b) treatment, and (c) day of follow-up.

For the analysis of P bioavailability, averages over time were taken for each of the biochar types according to the type of soil contaminant. For soils contaminated with Cd and Pb, it was not found that each type of biochar had a different significant effect on the bioavailability of P in the soil, leading to the conclusion that the type of biomass is not significant. For Cr-contaminated soils, at least one type of biochar was found to have a significantly different effect on the soil P bioavailability. It was found that the P bioavailability of sample 1.3 (BSAC + Cr) was 83.09% higher than that of sample 1.2 (BAC + Cr). It should also be noted that the lowest bioavailability of P in soil was recorded in sample 1.2 (BAC + Cr). This may explain why Cr bioavailability was recorded in sample 1.2 (BAC + Cr) at the end of the test, since Cr is an analog of P and a lower bioavailability of P in the soil may indicate that it is in the biochar competing for binding sites.^58,59^ However, to further elaborate on the above, a daily analysis of P bioavailability monitoring was performed for each soil type (Cr, Cd, or Pb) regardless of the type of treatment (Figure 4c). No significant effect of time on P bioavailability was found for the three soil types. However, in the case of Cd, it is possible to see how the bioavailability of P in the soil increases over time, indicating that the process of P release is generated and consequently favors the decrease of bioavailable Cd in the soil. Contrary to the case of Cd soil, in Pb soil P bioavailability decreased as Pb bioavailability decreased. The above suggests that the decrease in bioavailable Pb may be related to the increase in pH, as argued by Lebrun et al.^50^

P bioavailability analysis was also performed by the HM group, and no significant difference in P bioavailability was found among the three types of HM. However, it can be noted that in general the bioavailability of P was lower in the soil with Cr than in the other soil types (Figure 4a). Grouping by biochar type (Figure 4b) did not show that biochar had a significantly different effect on P bioavailability.

#### 2.1.5 Correlation Analysis of Variables

Correlation analysis was performed using Spearman’s Rho coefficient between the variables followed over time in the study: pH, bioavailable P, bioavailability of HMs, and EC (Table 1). Based on this, a significant and negative correlation was found between pH and P bioavailability (ρ = −0.280), a positive and significant correlation between pH and EC (ρ = −0.556), and a positive and significant correlation between EC and HMs bioavailability (ρ = −0.260).

**Table 1:** Correlation Coefficients between pH, Bioavailable P, and Bioavailability of HMs and EC

	pH	available P	HMs	EC
pH	1			
available P	–0.280^a^	1		
HMs	0.173	–0.109	1	
EC	0.556^a^	–0.222	0.260^a^	1

a *p* < 0.05.

### 2.2 Physico-Chemical Properties of the Land before and after a Phytoremediation

#### 2.2.1 Water Holding Capacity (WHC)

**Figure 5:**
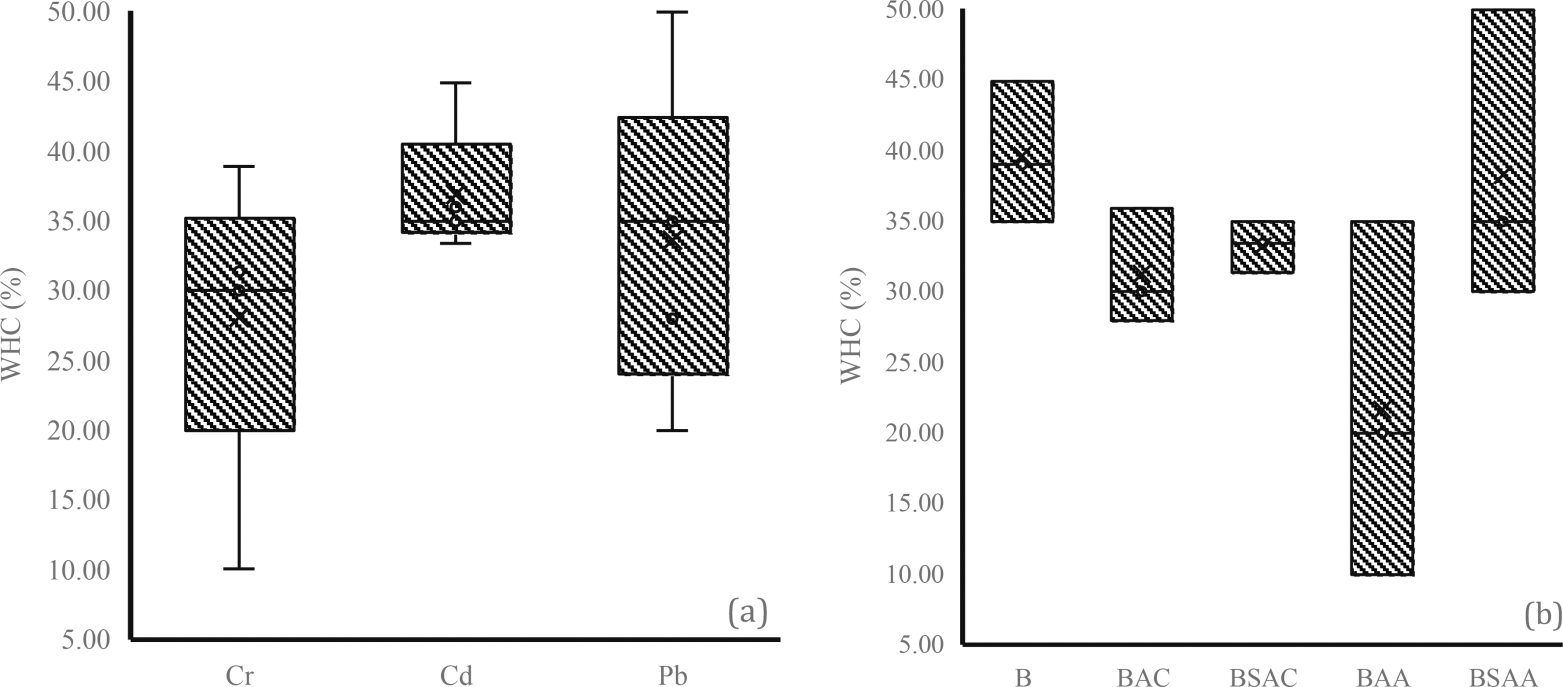
Water holding capacity (WHC) values grouped by (a) HM group and (b) biochar group.

For WHC analysis, the mean value per HM group (Figure 5a) and per biochar group (Figure 5b) was compared. When analyzed by contaminant, no HM was found to have a significantly different effect on the WHC of the samples. Analysis by biochar type showed that no biochar had a significantly different effect on the WHC of the samples.

WHC was also compared between the control (without biochar) and the other samples (with biochar), and no significant difference was observed between them. Although the difference was not significant, it is important to note that, contrary to initial expectations that the addition of biochar would improve WHC due to its high absorptive capacity, porous structure, and increased soil aggregation,^69^ overall, the controls (without biochar) showed 27.33% higher WHC than the other samples (with biochar). Consistent with the study by Gray et al.,^70^ the absence of effect of biochar on soil WHC was related to its hydrophobicity. The degree of hydrophobicity of a biochar depends on its composition: those with low hydrogen and oxygen content usually have high hydrophobicity, and the presence of an abundance of aromatic compounds on their surface increases their hydrophobic character.^71^ This hydrophobicity creates a negative capillary force, which prevents the infiltration of water into the pores of the biochar.^70,72^ However, the WHC of samples 2.2 (BAC + Cd) and 3.5 (BSAA + Pb) was higher with respect to the initial soil WHC, with a percentage increase of 2.8% and 42.93%, respectively, while sample 3.5 showed 42.94% more WHC compared to its control (nonbiochar control + Pb).

#### 2.2.2 Moisture Content

**Figure 6:**
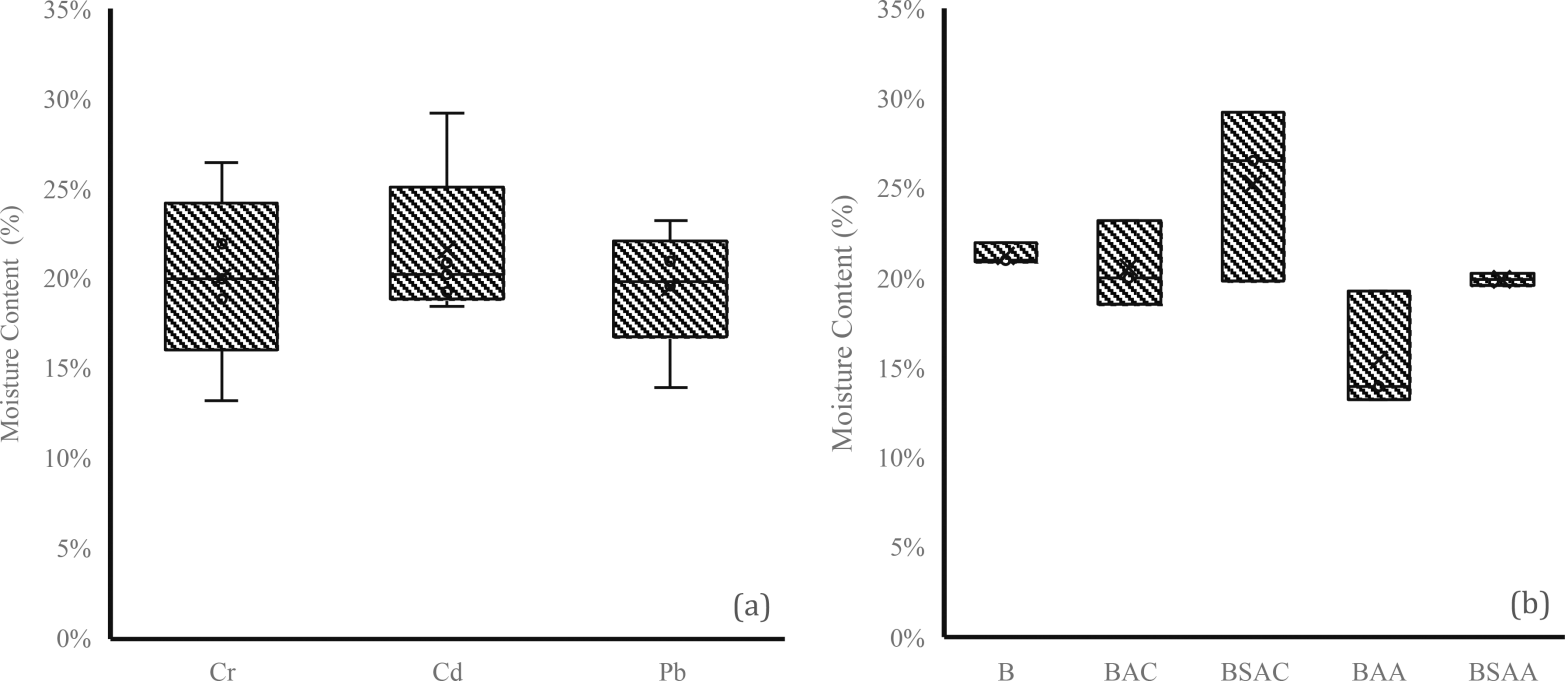
Moisture content values grouped by (a) HM group and (b) biochar group.

For moisture content analysis, the mean value per HM group and per biochar group was compared (Figure 6). Analysis by HMs showed that no HM had a significantly different effect on the moisture content of the samples. Analysis by biochar type showed that at least one type of biochar had an effect. It was determined that the moisture of the samples with BAA was significantly different from the samples with BSAA, being 62.83% higher in the former. It should be noted that the moisture content of sample 2.4 was 51.82% higher than the initial moisture content of the soil (Figure 6).

#### 2.2.3 Total Nitrogen Kjeldahl

**Figure 7:**
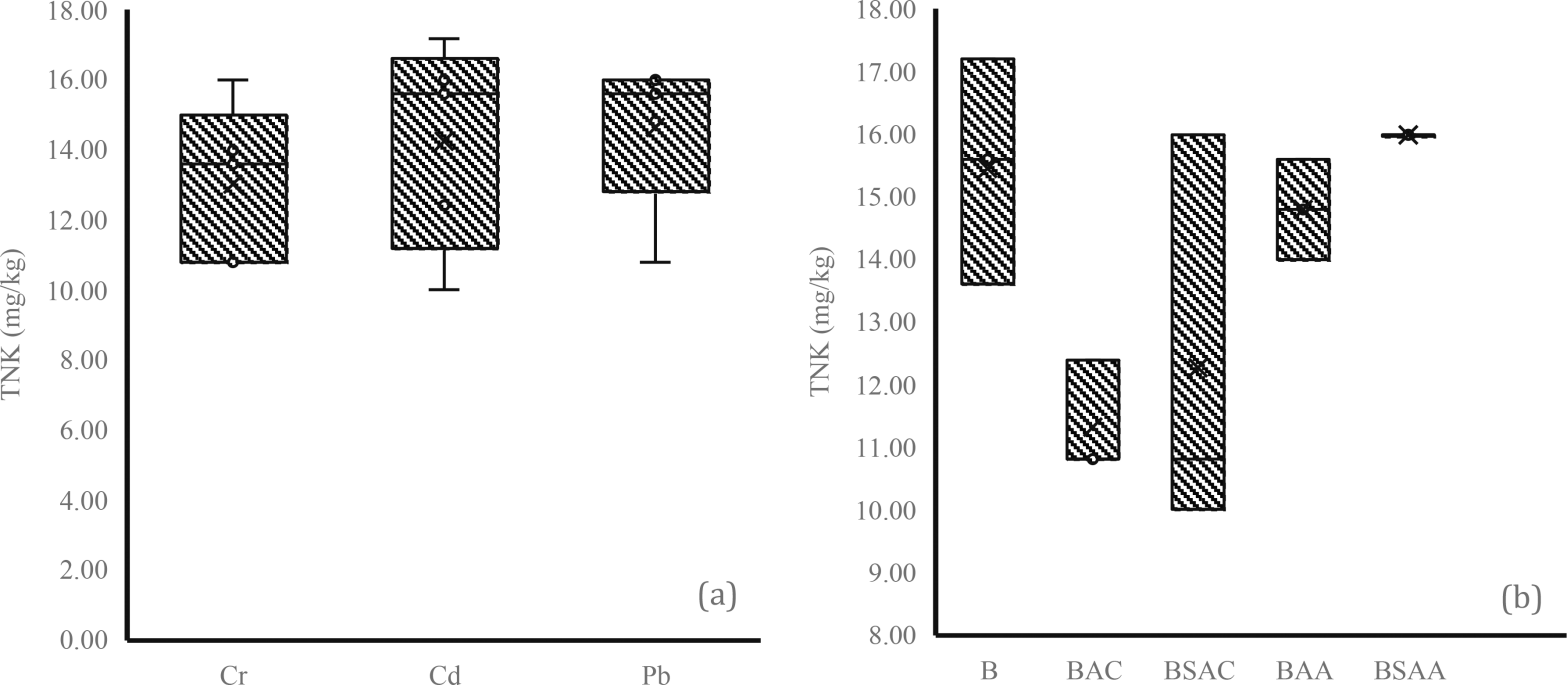
TNK values grouped by (a) HM group and (b) biochar group.

For the analysis of total Kjeldahl nitrogen (TNK) concentration, the mean was compared by the HM group (Figure 7a) and by the biochar group (Figure 7b). When analyzed by contaminant, no HM was found to have a significantly different effect on the TNK concentration of the samples. Analysis by biochar type showed that at least one type of biochar had a significantly different effect on the TNK concentration of the samples. It was determined that the TNK concentration of the samples with BSAA was significantly different from the samples with BAC, the TNK concentration being 42.85% higher in the samples with BSAA with respect to BAC.

The TNK concentration was also compared between the control (without biochar) and the other samples (with biochar), and no significant difference was observed between them. Although the difference was not significant, it is worth mentioning that the TNK concentration of the control was 13.72% higher with respect to the other samples. This may be because nutrients released into the soil pore water may leach out and be lost. In addition, biochar can reduce nutrient availability due to its high sorption capacity. Several studies have analyzed the sorption properties of biochar and observed that it can adsorb nitrate, ammonium, and phosphate,^73,74^ which reduces their leaching but also makes them less available to plants.^2,50^

It is important to note that the final TNK concentrations of all samples were lower than the initial TNK concentrations of the soil. This decrease in TNK content could be related to the fact that the comparison was made between the initial and final concentration, the decrease in TNK content could be due to the own nutrient consumption of *L. perenne* plants over time.

#### 2.2.4 Cation Exchange Capacity (CEC)

**Figure 8:**
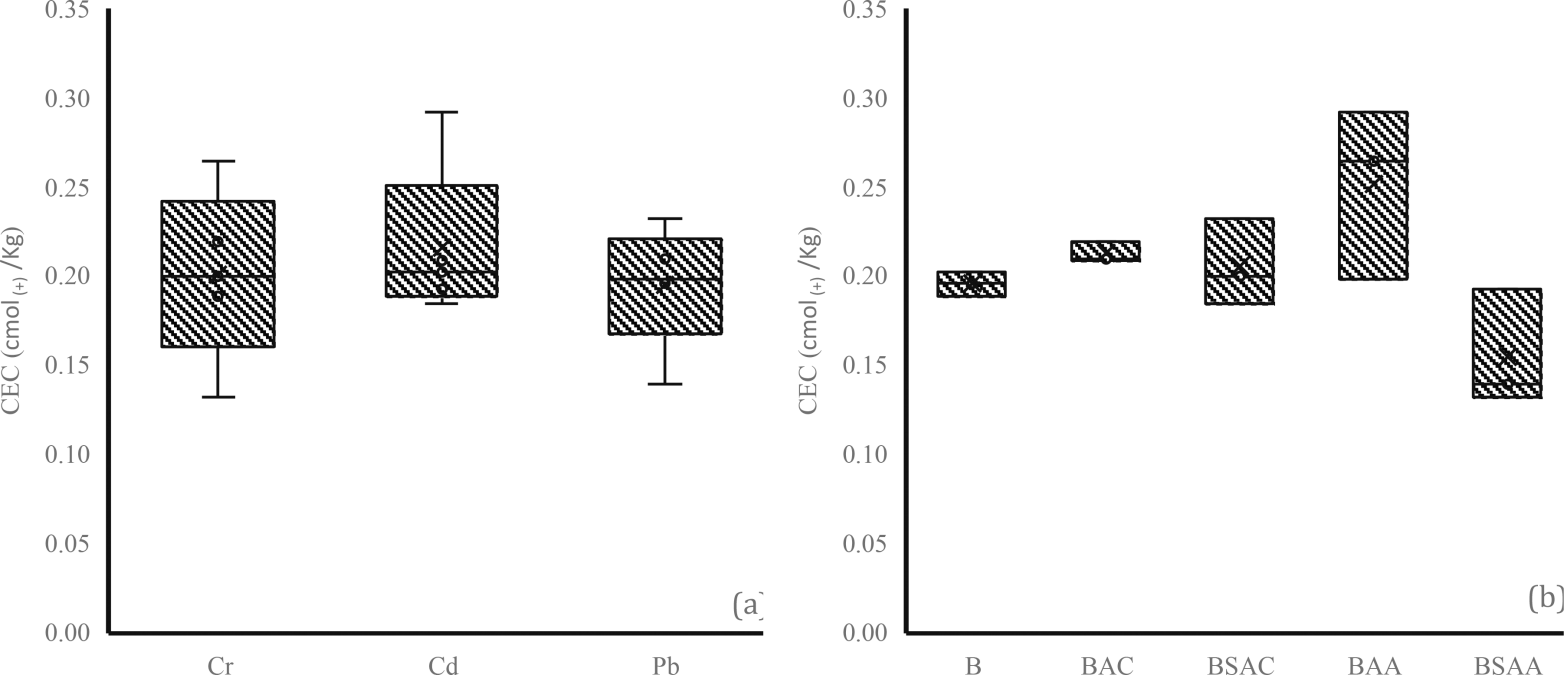
CEC values grouped by (a) HM type and (b) biochar type.

For CEC analysis, the mean value per HM group (Figure 8a) and per biochar group (Figure 8b) was compared. When analyzed by contaminant, no HM was found to have a significantly different effect on the cation exchange capacity of the samples. When the samples were analyzed by biochar type, it was found that no biochar had a significantly different effect on the CEC of the samples. CEC was also compared between the control (without biochar) and the other samples (with biochar), and no significant difference was observed between them. Although this difference was not significant, it is worth noting that the CEC of the control was 37.11% higher than that of the others. This could be due to the relatively low CEC of the biochar applied, as biochar produced at low temperatures generally has a low CEC.^75^

#### 2.2.5 Organic Matter (OM) Content

**Figure 9:**
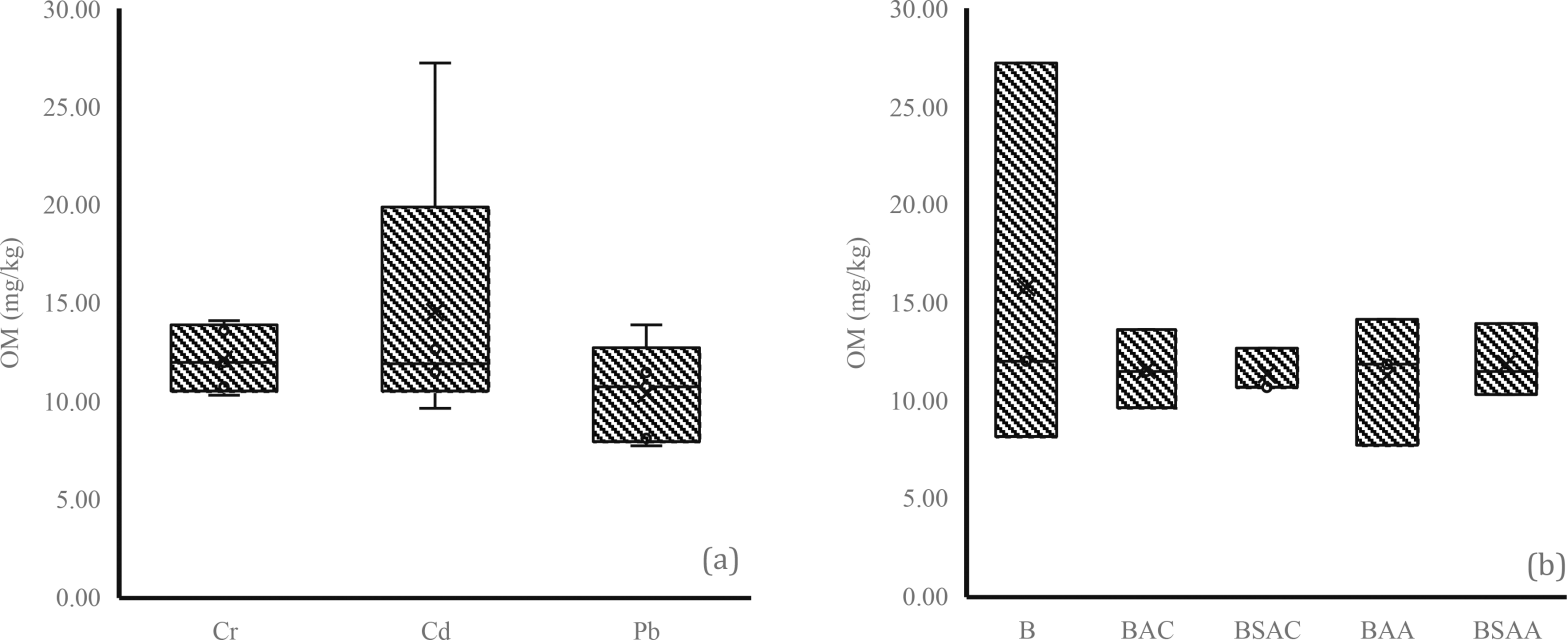
Organic matter content values grouped by (a) HM type and (b) biochar type.

For the analysis of OM content (carbon concentration), the mean value per HM group (Figure 9a) and per biochar group (Figure 9b) was compared. When analyzed by contaminant, it was found that no HM had a significantly different effect on the OM content of the samples. Analysis by biochar type showed that no biochar had a significantly different effect on the OM content of the samples. However, it can be observed that in all samples except sample 3.1 (nonbiochar control + Pb) and sample 3.4 (BAA + Pb), an increase in carbon concentration can be observed with respect to the initial carbon concentration. In the case of samples with biochar, the increase may have occurred due to the high carbon and OM content of biochar, which resulted in an increase in the organic carbon and OM content of the contaminated soils.^75^ This is beneficial because organic matter serves multiple functions in the soil; it contributes to soil aeration and promotes water and nutrient retention. It also provides essential substrates for soil microorganisms.^76^ Instead, a soil with a higher OM content has a greater capacity to retain metals.^77^ The decrease in OM content in samples 3.1 and 3.4 could be related to an improvement in microbial activity that induces the degradation of organic carbon.^50,78^

### 2.3 Heavy Metal (HM) Immobilization in the Plant

#### 2.3.1 Plant Organ Length

**Figure 10:**
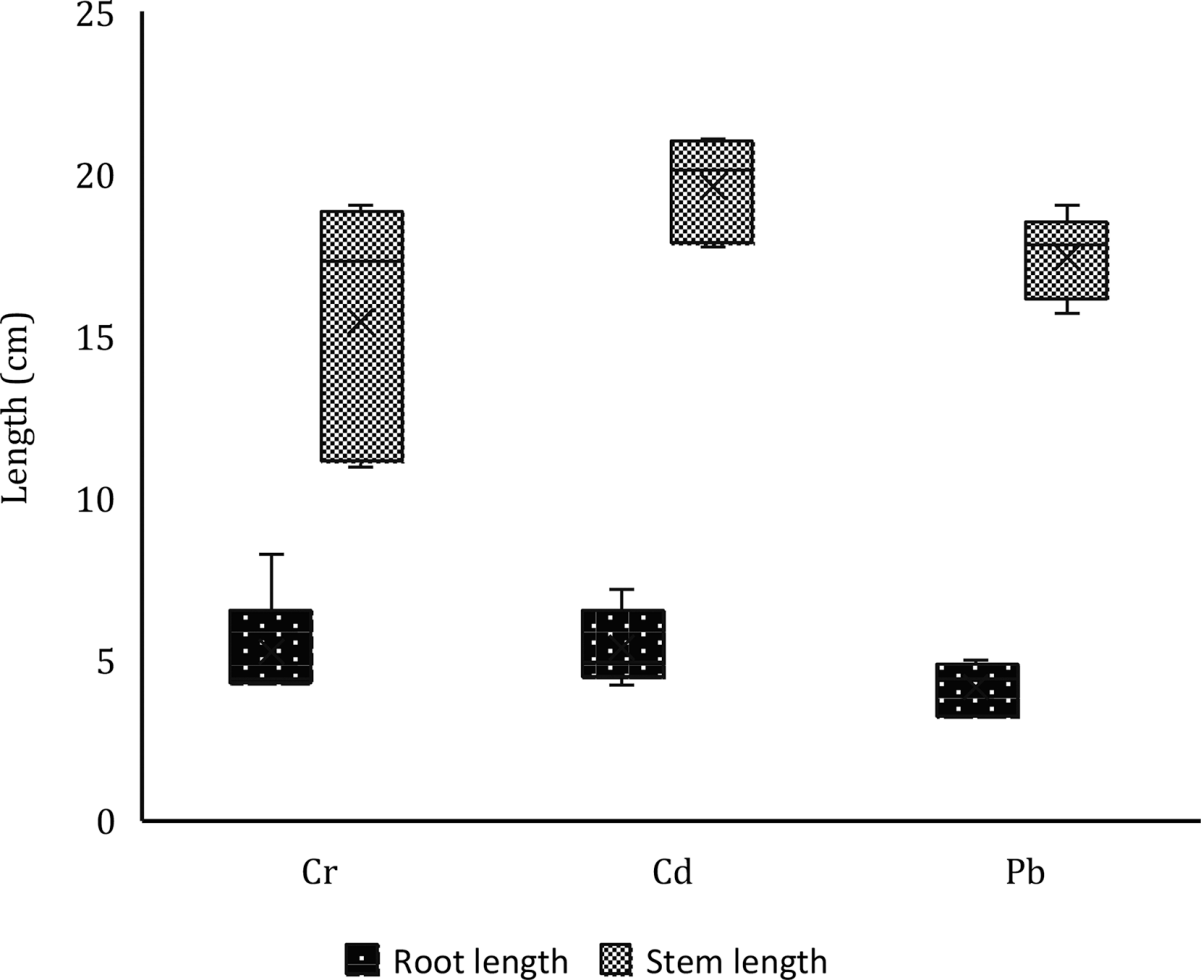
Effect of heavy metal concentration on the final size of the plant organs (root and stem)

The final length of the plant organs on day 45 is shown in Figure 10. It has been observed that the average length of the stems is greater than that of the roots. The average final length of the seedlings was 22.87 cm. A comparison was also made between the heights of seedlings planted in the control (without biochar) and seedlings planted in the other samples (with biochar).

It was also decided to compare the length of plant organs by the HM group, and it was found that the type of HM had no significant effect on the height of seedlings. The analysis was done by the biochar group, and it was found that the type of biochar also had no significant effect on seedling length. Therefore, it can be concluded that seedling growth was similar in all experiments.

#### 2.3.2 Metal Concentrations in Plant Organs

**Figure 11:**
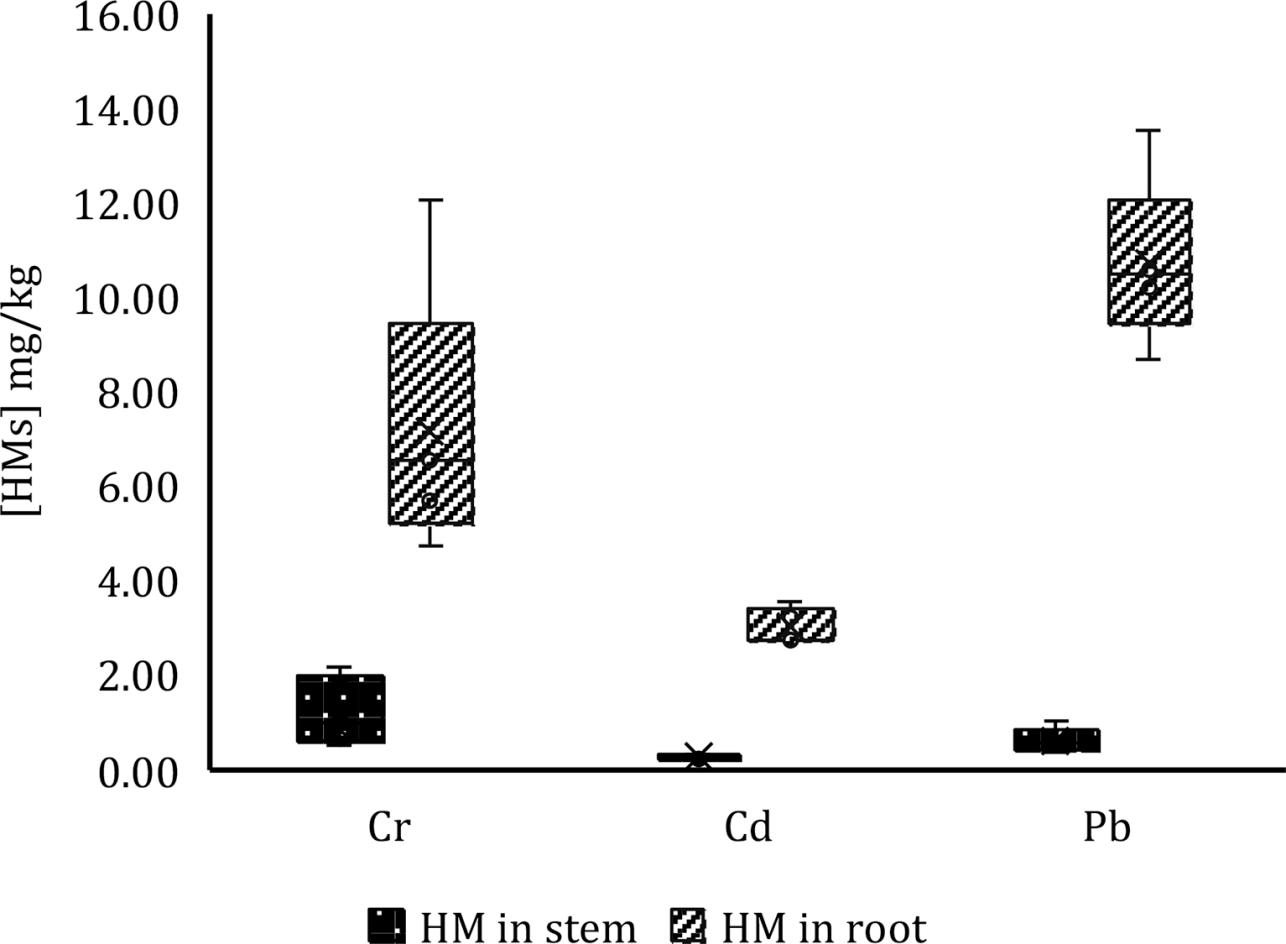
HM distribution in plant organs.

The difference in HMs concentration between the stem (plant area) and the root was compared, and significant differences were found between both groups for each of the soils contaminated with Cr, Cd, and Pb (Figure 11). The analysis of Cr bioavailability of stems and roots found in the Cr-contaminated soil revealed a significant difference between roots and stems, with the concentration in roots being 83.00% higher than in stems. Instead, the analysis of the Cd bioavailability of the stems and roots found in the soil contaminated with Cd revealed a significant difference between the two groups, with the concentration in the roots being 91.02% higher than in the stems. Finally, Pb bioavailability analysis of stems and roots found in Pb-contaminated soil indicated a significant difference between the two groups, with the concentration in roots being 94.98% higher than in stems. Therefore, it can be concluded that *L. perenne* favors more HM retention in roots than in stems. Figure 11 shows the distribution of the HM concentration in plant organs.

A comparison was also made between the HM concentrations of the plants sown in the control (without biochar) and the plants planted in the other samples (with biochar). It has been found that the effect of the type of biochar was not significant on the concentration of HM retained in the plant organs. However, it can be highlighted that plant organs in soils with biochar had 11.6% more HM retention. These results coincide with those reported in the literature, in which the addition of biochar to the soil can modify soil properties such as pH and EC.^42,43,64^

The concentration of HM retained in plant organs by the HM group was also compared. A marginally significant difference was found in the effect of the HM type on the concentration of HM in plant organs. A separation was made between root and stem by HM group, and the following was evidenced: (i) This significant difference was observed between the concentration of HM in the stem with Cd and Cr, the concentration of Cr being 51.85% higher than that of Cd. The lowest concentration was that of Cd in the stem. (ii) The type of HM was found to have a significant effect on the HM concentrations retained in the root. It was shown that all of the HM concentrations in the root were different from each other; therefore, on average, Pb was the most retained by the root, followed by Cr and finally Cd.

**Figure 12:**
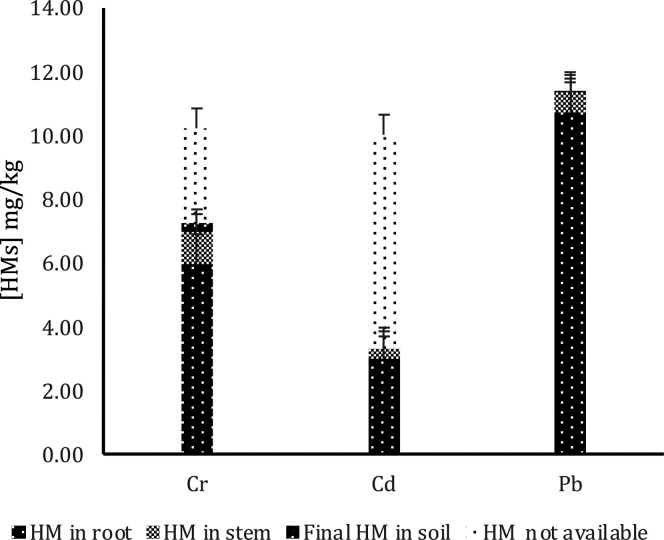
Balance of HM concentration is the soil–plant system.

The results of this work may suggest that, depending on the type of metal, the plant uses a remediation mechanism. These findings align with other studies where *L. perenne* has been employed as a phytoremediation plant. Previous authors have demonstrated that *L. perenne* exhibits metabolic changes when exposed to Cd^2+^ concentrations of 0.25 mg/L, while no adverse effects were observed at concentrations exceeding 25 mg/L Cd^2+^.^60^ This observation might explain one of the primary reasons for the current study, where Cd was among the metals found in the plant in the lowest proportion and in an available form. Figure 12 explains a mass balance that considers the initial metal concentration in the soil and compares it with the values obtained from the ICP-OES analysis. This balance indicates that, for Cd and Cr, a significant percentage of the metals are not bioavailable in either the plants or the soil. Contrarily, in the case of Pb, more than 90% of it is bioavailable in plants.^30,60,61^ The low absorption of Cd by *L. perenne* has been reported in the literature, as shown in the results of the present work. Then, the low bioavailability present in this study could be attributed to the biochar applied to the soil, which is possibly retaining the Cd and not leaving it trapped in its structure and not available for the plant.^30,60,61^ The behavior depicted in Figure 12 has been observed in similar studies, demonstrating *L. perenne*’s capability to absorb metals like Pb and Hg, although in low quantities for Cd. The bioavailability of these metals is contingent upon the plant’s unique absorption mechanisms for each metal.^30,61^ Regarding Cr, existing literature demonstrates phytoimmobilization mechanisms, primarily in the roots, where processes such as sorption, precipitation, complexation, or alteration of the metal’s valence can occur within the rhizosphere.^79^

## 3 Conclusions

It can be concluded that the application of the four types of biochar, especially activated biochar, enriched the physical properties of the soil, such as pH and EC. No significant effect of the type of biochar on the bioavailability of HMs has been found. However, low levels of HM bioavailability have been observed in the soil (<1 mg/kg). The bioavailability of Cr increased but remained at a low level. This behavior can be attributed to the lower bioavailability of P. This is because Cr is a P analogue, and therefore lower P bioavailability in soil may indicate competition for binding sites on biochar.

It is for this reason that when soils are contaminated with Cr, special care must be taken when adding P-laden amendments, especially biochar. The addition of biochar was found to have no significant effect on the concentration of HM retained in plant organs or on the bioavailability of HM in soils. However, it should be noted that the HM retention was 11.6% higher in plant organs found in soils with biochar.

Since heavy metal generally has low bioavailability in soil, it can be concluded that biochar-assisted phytoremediation has been successful. This study has shown that the application of amendments such as biochar to soil in different forms, such as activated biochar and P-loaded biochar, can promote phytoremediation of soil metals. All the tests applied in this study have demonstrated high removal efficiencies of Cd, Cr, and Pb in assisted phytoremediation processes.

## 4 Materials and Methods

### 4.1 Soil Preparation

A soil sample was taken from the north side of the Universidad de Los Andes, Bogotá, Colombia. Its particle size after sieving was 2 mm. A soil fraction (300 g) was extracted for initial analyses of texture based on the Bouyoucos method, with the pH based on EPA SW-846 9045 D-Revision 4-2004, EC based on SM 2510 B Edition No.22-2012, WHC (NTC 5167 standard), OM content based on the Walkley–Black standard method, TNK content according to SM 4500-Norg-C, and SM 4500-NH_3_ B and C modified and based on ISO 11261:1995 (E), with CEC in the NTC 5167 standard, available phosphorus extraction based on the OLSEN method, quantification done by colorimetry (Standard Methods, SM-4500-P C), and determined by moisture by thermobalance.

A 0.01 M CaCl_2_ solution was employed to assess Cr, Cd, and Pb bioavailability, extracting water-soluble and easily exchangeable metals from soil samples.^32^ The CaCl_2_–Pb extraction method utilized a soil-to-extractant ratio of 1:5. Soil was mixed with the 0.01 M CaCl_2_ solution and agitated at 100 rpm for 2 h, followed by the separation of soil via filtration (0.45 μm).^80^

The levels of Cd, Cr, and Pb in the supernatants were measured using ICP-OES (ICP-OES Thermo Scientific ICAP6500 DUO equipment, Thermo Scientific, Waltham, USA). This involved conducting microwave digestion according to the EPA-3051A method, followed by ICP-OES analysis utilizing the EPA-6010D method to obtain readings.^32,81^ The initial physicochemical properties of the soil can be seen in Table 2. For soil contamination, the soil was divided into three equal parts; each part was impregnated with each of the three metal solutions (Cd 10 mg/kg–Cr 10 mg/kg–Pb 10 mg/kg), completely homogenized, and finally left to stand for 7 days.

**Table 2:** Physico-Chemical Properties of the Initial Soil^a^

physicochemical parameter	initial soil
texture	sandy-loamy
pH	5.44
EC (μS/cm^–1^)	364.00
WHC (%)	34.93
CEC (c_mol_^(+)^/kg)	2.30
OM (mg/kg)	8.89
TNK (mg/kg)	11.79
P (mg/kg)	171.01
H (%)	19.25%
[Cd] (mg/kg)	0.02
[Cr] (mg/kg)	0.20
[Pb] (mg/kg)	0.18

a EC, electrical conductivity; WHC, water holding capacity; CEC, cation exchange capacity; OM, organic matter; TNK, total Kjeldahl nitrogen; P, available phosphorus; H, humidity; [Cd], available cadmium; [Cr], available chromium; [Pb], available lead.

### 4.2 Biochar Production

Biochar was obtained from two types of residues present in the agricultural industry: coffee husks and sugar cane leaf. For this purpose, the material was prepared by drying and grinding the biomasses. The prepared total biomass samples were subjected to a pyrolysis process at 500 °C with a residence time of 10–15 min in accordance with previous works published by the research group and in which a methodology has been developed to obtain biochar.^32,82,83^ On the other hand, half of the material was activated with KOH in a 1:1 ratio, left in contact for a period of 24 h, and then thermally activated at a temperature of 500 °C with a heating rate of 5 °C/min and a residence time of 2 h.^32,82−86^ The resulting biochar was impregnated with 4000 mg/L potassium phosphate to mirror saturation, left in contact for 45 min, filtered, and dried at 60 °C for 24 h.^32^ Four types of biochar were obtained from the above process: activated coffee husk biochar (BAC), non-activated coffee husk biochar (BSAC), activated sugar cane leaf biochar (BAA), and nonactivated sugar cane leaf biochar (BSAA). All experiments were performed in triplicate in order to have statistical significance. The analysis of pore size (Table 3) involved conducting a BET area analysis using AutoChem II 2920 equipment (Micromeritics, Norcross, USA). All biochar samples exhibit two pore types: micropores and larger average pores, with micropores measuring 2 nm in size. Table 3 shows the main properties evaluated for biochar. The significant improvement in both the carbon content and the surface area of the coals after being activated can be highlighted. These results coincide with other literature.^83,85,87^

**Table 3:** Characterization of Activated and Nonactivated Biochar

		coffee husk		sugar cane leaf	
parameter		activated	nonactivated	activated	nonactivated
superficial area S (m^2^/g)		622	454	405	338
elemental analysis	nitrogen (%)	1.87	0.85	0.51	0.73
	carbon (%)	54.4	42.0	36.4	24.9
	sulfur (%)	0.38	0.17	0.19	0.29
	hydrogen (%)	1.09	4.13	3.48	0.48
	oxygen (%)	42.31	52.90	59.44	73.60
high heat value (HHV; MJ/kg)		19.46	18.00	14.93	9.21

To generate P-loaded biochars, the procedure involved immersing both raw biochar and activated biochar in a saturated KH_2_PO_4_ solution, following the method outlined in Sepúlveda-Cadavid et al.^88^ The biochar was subsequently separated through filtration using a 0.45 μm membrane, followed by cleaning with distilled water, drying at 60 °C, and eventual storage. Determination of the bioavailable P content was performed using the Olsen method (Olsen-P).^32,88^ In this method, 1.0 g of solid material was combined with 20 mL of 0.5 M NaHCO_3_ solution (pH 8.5) and agitated at 100 rpm for 30 min. The solid residue was then separated via filtration (0.45 μm).

### 4.3 Experimental Setup

According to previously published work, 1% of each biochar plus a nonbiochar control was added to each contaminated soil.^25,32^ Subsequently, the experiment has been carried out in a rhizobox, which was filled up to 1 cm below the border with soil (approximately 1 kg) plus its corresponding biochar. Experiments were carried out in duplicate, in which 50 seedlings of *L. perenne* were sown in duplicate.^60−62^ The experimental setups were irrigated with deionized water twice a week. In total, 12 samples were obtained as shown in Table 4.

**Table 4:** Experimental Setup Code

sample	description
1.1	nonbiochar blank + Cr
1.2	soil with Cr + activated coffee husk biochar
1.3	soil with Cr + non- activated coffee husk biochar
1.4	soil with Cr + activated sugar cane leaf biochar
1.5	soil with Cr + nonactivated sugar cane leaf biochar
2.1	nonbiochar blank + Cd
2.2	soil with Cd + activated coffee husk biochar
2.3	soil with Cd + non- activated coffee husk biochar
2.4	soil with Cd + activated sugar cane leaf biochar
2.5	soil with Cd + nonactivated sugar cane leaf biochar
3.1	nonbiochar blank + Pb
3.2	soil with Pb + activated coffee husk biochar
3.3	soil with Pb + non- activated coffee husk biochar
3.4	soil with Pb + activated sugar cane leaf biochar
3.5	soil with Pb + nonactivated sugar cane leaf biochar

After being sown, seedlings were evaluated at 15, 30, and 45 days, measuring parameters such as pH, EC, phosphorus, and metal bioavailability. On day 45, seedlings were harvested, wet weight was determined, and both stem and root lengths were measured. They were then dried at 60 °C for 24 h and weighed. Then, the stem and root were separated, and the bioavailability of HMs of each plant organ was determined. Finally, the soil of each experimental setup was characterized and texture, WHR, OM, TNK, CEC, and moisture were measured according to the methods described in section 4.1.

### 4.4 Statistical Analysis

All statistical analyses were performed with StataSE 17 software. Mean values were compared using the parametric ANOVA test for normal data or the nonparametric Kruskal test for non-normal data. A posthoc test (Bonferroni or pairwise Kruskal test, respectively) was then performed. Statistical significance was set at *p* ≤ 0.05. Spearman’s Rho correlation analysis was used for correlation analysis.
